# Field-Deployable Detection of Chestnut Blight Pathogen *Cryphonectria parasitica* Using Enzyme-Mediated Duplex Exponential Amplification

**DOI:** 10.3390/cimb47090762

**Published:** 2025-09-15

**Authors:** Shuai Wang, Zhongwei Feng, Yiming Liu, Changyun Tang, Kai Guo, Jiafu Hu

**Affiliations:** 1 School of Forestry and Biotechnology, Zhejiang A&F University, Hangzhou 311300, China; 2 Suichang County Ecological Forestry Development Center, Lishui 323300, China

**Keywords:** chestnut blight, *Cryphonectria parasitica*, enzyme-mediated duplex exponential amplification, field diagnosis

## Abstract

Chestnut blight caused by *Cryphonectria parasitica* poses a major threat to chestnut plantations worldwide. Rapid, field-deployable diagnostic tools are essential for effective disease surveillance and management. Here, we developed and validated an enzyme-mediated duplex exponential amplification (EmDEA) assay for the specific and sensitive detection of *C. parasitica*. The assay successfully distinguished *C. parasitica* from non-target fungal species with a detection limit of 10 pg of genomic DNA per reaction. Application to naturally infected bark samples yielded results consistent with those of conventional qPCR. The complete workflow, including crude DNA extraction and fluorescence-based detection under isothermal conditions, was completed within 35 min. Our findings demonstrate that the EmDEA assay is a sensitive, robust, and field-adaptable tool that can be used for the early detection of chestnut blight, with significant potential for deployment in resource-limited environments.

## 1. Introduction

Chestnut blight, caused by the ascomycete fungus *Cryphonectria parasitica* (Murrill) Barr (Cryphonectriaceae, Diaporthales), is a devastating disease of the chestnut *Castanea mollissima*. *C. parasitica* was first reported in North America in 1904, where it nearly eradicated the American chestnut within several decades [1]. The pathogen exhibits a broad host range, infecting not only *Castanea* species but also *Quercus*, *Acer*, and *Eucalyptus* spp. [2,3]. Infection typically occurs through bark wounds, where small, water-soaked, circular lesions develop, later desiccating and cracking, eventually girdling the cambium and exposing the underlying wood. In older trees with thicker bark, symptoms are often less conspicuous, delaying timely diagnosis and intervention [4].

Although both morphological and molecular diagnostic tools have advanced significantly, most are not suited for rapid, accurate, and low-cost detection in field settings. Morphological identification requires fungal isolation and microscopic examination of reproductive structures, which is time-consuming and requires taxonomic expertise and lab infrastructure. Molecular approaches, including conventional PCR, DNA fingerprinting, restriction fragment length polymorphisms (RFLPs), random amplified polymorphic DNA (RAPDs), nested PCR, and real-time PCR, have greatly enhanced diagnostic speed and specificity [5,6,7,8,9,10,11,12,13]. However, these conventional methods remain largely confined to laboratory use due to thermal cycling requirements, long reaction times, and reliance on sophisticated instruments and trained personnel.

Among isothermal methods, LAMP is one of the most widely adopted due to its high amplification efficiency and specificity under constant-temperature conditions. However, LAMP requires careful primer design involving four to six primers and relatively high reaction temperatures (~60–65 °C), and it often generates non-specific amplification products that may complicate interpretation in field use. In addition, post-reaction handling is sometimes necessary, which increases the risk of contamination [14].

To overcome these limitations, we explored enzyme-mediated duplex exponential amplification (EmDEA), a relatively new isothermal amplification technology. EmDEA integrates target amplification and signal amplification into a single closed-tube system, enabling each target molecule to generate thousands of fluorescent signals, thereby achieving high sensitivity. Unlike LAMP, the reaction operates at a lower constant temperature (42 °C), requires only one pair of primers, and is completed within approximately 35 min. The assay is simple to perform: operators with minimal training can initiate the test by adding the DNA sample and a single reaction activator, without complex pipetting or multiple preparation steps. Furthermore, a built-in nuclease degrades nucleic acids at the end of the reaction, reducing the risk of carryover contamination and false-positive results. These characteristics make EmDEA a promising alternative to LAMP for rapid, accurate, and on-site detection of *C. parasitica.*

This study evaluated the performance of an EmDEA-based platform for detecting *C. parasitica*. Primers and probes targeting the translation elongation factor-1α (*TEF-1α*) gene were designed and screened, and the sensitivity, specificity, and suitability of the assay for rapid on-site detection were systematically assessed.

## 2. Materials and Methods

### 2.1. Sample Sources

A total of 28 strains were tested. Strain Cp1, a standard isolate of *C. parasitica* (CFCC52150), was provided by Prof. Chengming Tian’s research group at the Department of Forest Protection, Beijing Forestry University (China). Cj, a standard isolate of *C. japonica* (CFCC 52148), was obtained from the China Forestry Microbial Culture Collection Center. The remaining strains were obtained from the Forest Protection Laboratory of Zhejiang Agriculture and Forestry University (China). All strains were cultured on potato dextrose agar (200 g potatoes, 20 g dextrose, 20 g agar per L) at 25 °C. Information regarding these strains is provided in Table 1.

Twelve phloem tissue samples exhibiting chestnut blight were collected from plantations in Taian, Shandong Province, and Lishui, Zhejiang Province (China). Following isolation, and morphological and molecular identification in our laboratory, all samples were confirmed to be infected with *C. parasitica.*

### 2.2. Genomic DNA Extraction from Strain Cultures and Infected Bark Tissues

The DNA of cultured strains was extracted using an improved cetyltrimethylammonium bromide (CTAB) method [15]. DNA yield and purity were assessed using a NanoDrop 2000 spectrophotometer (Thermo Fisher Scientific, Loughborough, UK). For infected bark tissue samples, genomic DNA was extracted using a commercial kit from Baichuan Biotechnology (Zhejiang, China), following the manufacturer’s instructions. Briefly, ~100 mg of symptomatic wood chips was scraped with a sterile blade into a 1.5 mL microcentrifuge tube, mixed with 1 mL of lysis buffer, and incubated at 95 °C for 5 min in a dry-block heater. A 50 µL aliquot of lysate was then diluted into 200 µL of pre-aliquoted dilution buffer in a 500 µL tube, and 50 µL of the resulting solution served as the template for subsequent isothermal fluorescence amplification.

### 2.3. Primers and Probes

DNA primers were designed based on the *C. parasitica TEF-1α* gene fragment available in the GenBank database [16]. Multiple *C. parasitica* isolates were aligned in MEGA11 to identify conserved intraspecific regions, while sequences from related fungal species were included to ensure interspecific specificity [17]. Conserved and species-specific regions were selected for primer and probe design. The forward primer contained a T7 promoter and was synthesized by Beijing Tsingke Biotech Co., Ltd. (Beijing, China). The RNA fluorescent probe was synthesized by Suzhou GeneVide Biotech Co., Ltd. (Suzhou, China) with a 6-carboxyfluorescein (FAM) fluorescent reporter group at the 5′ end and Black Hole Quencher 1 (BHQ1) quencher group at the 3′ end. The primer and probe sequences are provided in Table 2.

### 2.4. Isothermal Fluorescence Amplification Assay

**Figure 1:**
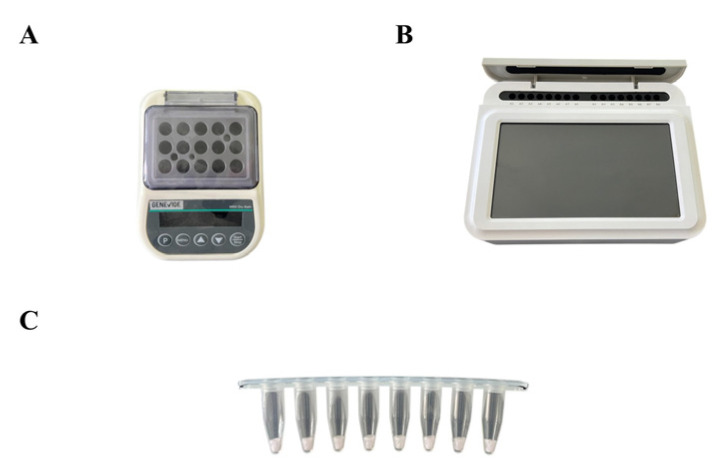
Portable EmDEA system. (**A**) Dry block heater; (**B**) portable fluorescence device; (**C**) lyophilized pellets in test tubes including primers and probes.

The EmDEA detection assay was conducted using an EmDEA test kit (Figure 1C) (GeneVide, Suzhou, China) according to the manufacturer’s protocol. The forward and reverse DNA primers (10 μM each) and RNA probe (250 ng/μL) were lyophilized in a single reaction pellet (freeze-dried ball) within the kit. For each 20 μL reaction, 5 μL of DNA template and 15 μL of activation solution were added directly to the freeze-dried pellet to rehydrate the reaction mixture, corresponding to 1 μL of each primer and 1 μL of RNA probe per reaction. Real-time amplification was performed on a qTOWER 2.0 instrument (Analytik Jena AG, Jena, Germany) at a constant 42 °C for 30 min, with fluorescence signals recorded every minute.

For field-applicable testing, a portable EmDEA system comprising a dry block heater and compact optical isothermal fluorescence device was provided by GeneVide Biotech Co., Ltd. The portable fluorescence device weighed <1 kg (Figure 1B), whereas the dry-block heater weighed < 0.7 kg (Figure 1A). The assay was conducted at 42 °C with a total reaction time of 20 min, enabling simultaneous processing of up to 16 samples. The portable optical isothermal device was operated using a pre-programmed protocol that required no manual configuration.

### 2.5. RNA Probe Screening

Genomic DNA from the Cp1 strain was used as a template to evaluate four primer–probe combinations (F2R2, F2R3, F3R3, F3R2) generated by cross-pairing forward primers F2/F3 with reverse primers R2/R3. Each primer–probe set was tested alongside a negative control. Probes showing efficient amplification in preliminary trials were selected for downstream validation. For each primer–probe set, six candidate RNA probes were tested in parallel alongside a negative control. Amplification performance was assessed quantitatively based on fluorescence signal intensity and time to peak signal, and the probe showing the earliest and strongest amplification was selected for downstream validation.

### 2.6. Primer–Probe Screening and Optimization

With probe RNA6 and forward primer F2 fixed, six downstream primers (R1–R6) were tested, and the optimal primer was selected. Subsequently, with RNA6 and the selected downstream primer fixed, six upstream primers (F1–F6) were screened. Each primer–probe set was evaluated with a negative control. To visualize primer–probe binding sites, the aligned target gene region from representative *C. parasitica* isolates was prepared using ESPript 3.0 [18].

### 2.7. Evaluation of EmDEA Assay Specificity

The specificity of the optimized primer–probe pair (RNA6F5R3) was evaluated using DNA from all test strains listed in Table 1. Negative controls were included in each run to confirm the absence of nonspecific amplification. For each strain, DNA was extracted from a single biological sample, and each sample was tested in three independent technical replicates to ensure reliability and reproducibility of the assay.

### 2.8. Evaluation of EmDEA Assay Sensitivity and Stability

The sensitivity and reproducibility of the EmDEA assay were evaluated using DNA from a single biological sample (Cp1 strain). Negative controls were included in each run to confirm the absence of nonspecific amplification. For each DNA concentration (10 ng, 1 ng, 100 pg, 10 pg, 1 pg, and 100 fg per reaction), the assay was performed in three independent technical replicates to ensure reliability and reproducibility of the results.

### 2.9. Field Evaluation of the EmDEA Assay

The validated RNA6F5R3 primer–probe set was applied to DNA extracted from phloem tissues of naturally infected chestnut trees. Cp1 strain DNA served as the positive control, and RNase-free water served as the negative control. Reactions were performed using both a real-time qPCR system and a portable optical isothermal device.

## 3. Results

### 3.1. Identification of RNA Probes

**Figure 2:**
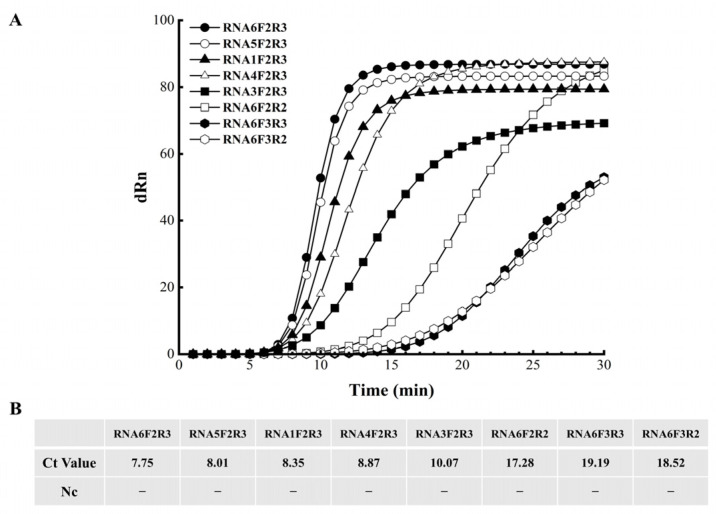
RNA probe screening. (**A**) Amplification curves for enzyme-mediated duplex exponential amplification (EmDEA); (**B**) cycle threshold (Ct) values. “Ct”: cycle threshold “−”: no Ct detected in negative control.

We evaluated the amplification performance of four primer pairs, each with six candidate probes. All primer pairs paired with probe RNA6 produced clear amplification curves, and RNA6 consistently generated earlier peaks and stronger fluorescence signals than the other probes (Figure 2A,B). A direct comparison of the four RNA6-based primer pairs revealed that F2/R3 produced the lowest Ct value and the strongest fluorescence signal, with no amplification observed in the negative control (Figure 2A,B). Consequently, RNA6–F2/R3 was adopted in all subsequent screening assays.

### 3.2. Optimization of Primer–Probe Combinations for EmDEA

**Figure 3:**
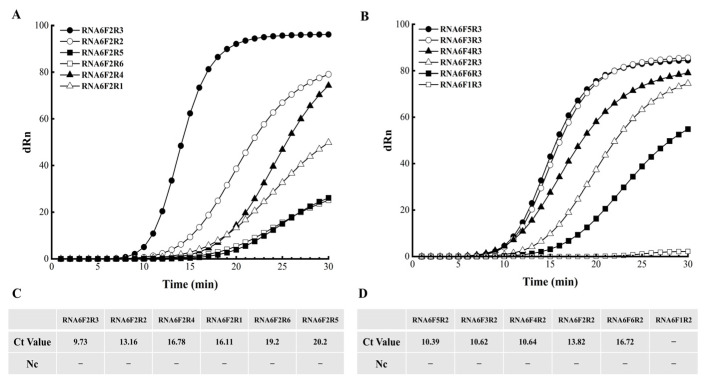
Screening and optimization of DNA primers for EmDEA. (**A**,**B**) Amplification curves for downstream and upstream primer candidates, respectively. (**C**,**D**) Summary tables of cycle threshold (Ct) values corresponding to each primer set. “Ct”: cycle threshold “−”: no Ct value detected in negative control.

Initially, the RNA6 probe was paired with the downstream primer F2 and tested against candidate primers R1–R6. Primer R3 had the lowest Ct and highest endpoint fluorescence, with no signal observed in the negative control; thus, it was selected as the optimal downstream primer (Figure 3A,C). Using RNA6 and R3, six upstream primers (F1–F6) were evaluated. Primer F5 had the lowest Ct value, with no amplification in the negative control, and was chosen as the optimal upstream primer (Figure 3B,D).

**Figure 4:**
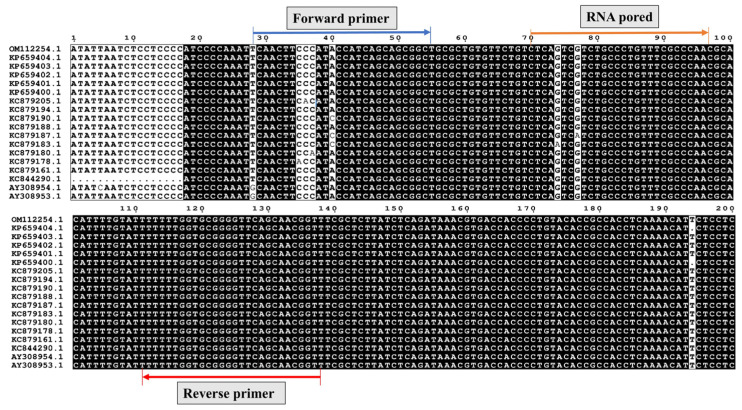
Multiple sequence alignment of the target region across representative *C. parasitica* isolates.

The finalized primer–probe set targets a 111-nucleotide region of the *TEF-1α* gene. Alignment of this region across representative *C. parasitica* isolates confirmed fully conserved binding sites for F5, R3, and RNA6, supporting the specificity and robustness of this primer–probe combination for reliable EmDEA detection (Figure 4).

### 3.3. EmDEA Assay Specificity

**Figure 5:**
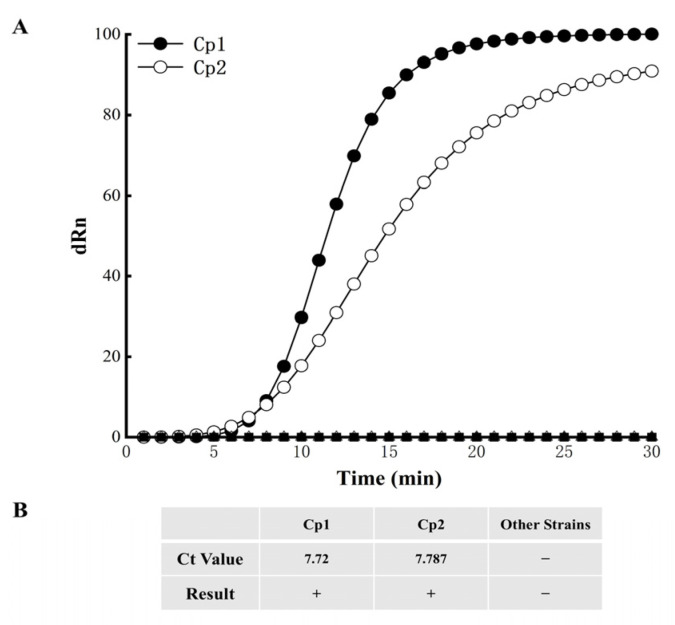
EmDEA assay specificity. (**A**) Amplification curves for EmDEA. (**B**) Ct values. “Ct”: cycle threshold; −: negative, +: positive. Representative data from three independent technical replicates are shown.

The specificity of the RNA6F5R3 primer–probe combination was assessed using DNA samples from two *C. parasitica* strains of distinct origins and other strains (see Section 2.7). Both *C. parasitica* strains yielded clear fluorescent signals, whereas no fluorescent signal was observed for any of the other strains (Figure 5A,B). These results were reproducible across three independent technical replicates, with *C. parasitica* consistently positive and all non-target strains consistently negative. These findings indicate that the RNA6F5R3 primer–probe combination exhibits high specificity, enabling accurate discrimination of *C. parasitica* from non-target strains.

### 3.4. EmDEA Assay Sensitivity

**Figure 6:**
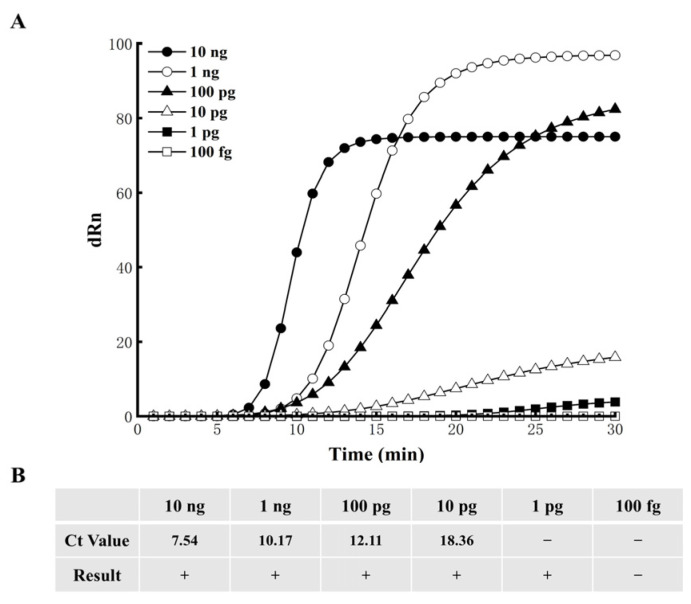
EmDEA assay sensitivity. (**A**) Amplification curves for one representative experiment. (**B**) Ct values for the corresponding experiment. “Ct”: cycle threshold; −: negative, +: positive. Representative data from three independent technical replicates are shown.

To assess the sensitivity, DNA from the Cp1 strain was serially diluted and used as a template for detection. The RNA6F5R3 primer–probe set was able to reliably detect DNA down to a dilution of 10^−3^, corresponding to a detection limit of 10.0 pg of total DNA per reaction. Across three independent technical replicates, each 10 pg DNA sample was consistently detected as positive, demonstrating the high sensitivity and reliability of the assay. Figure 6A,B show amplification curves and Ct values from one representative replicate.

### 3.5. Field Evaluation Results of the EmDEA Assay on Chestnut Blight Samples 

**Figure 7:**
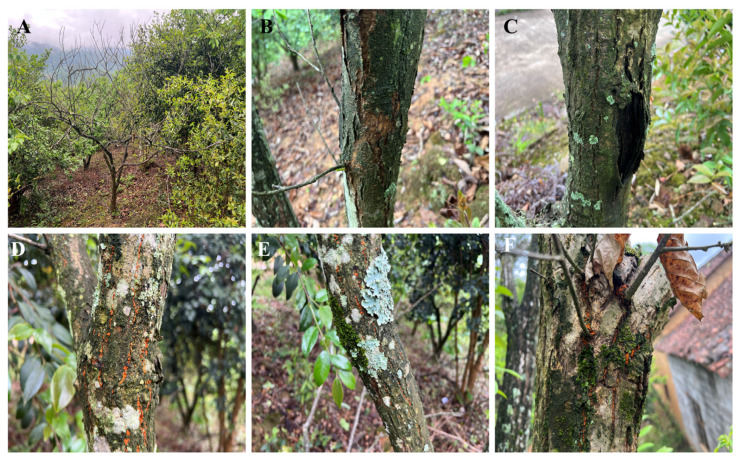
Castanea mollissima trees exhibiting symptoms of *C. parasitica* infection in a forest setting. (**A**) Castanea mollissima killed by *C. parasitica*. (**B**,**C**) Characteristic bark cracking and swelling on Castanea mollissima induced by *C. parasitica* infection. (**D**–**F**) On the infected bark, the fungus produces clusters of yellow-orange to reddish-brown pustules (stromata) that contain either sexual or asexual fruiting bodies.

**Figure 8:**
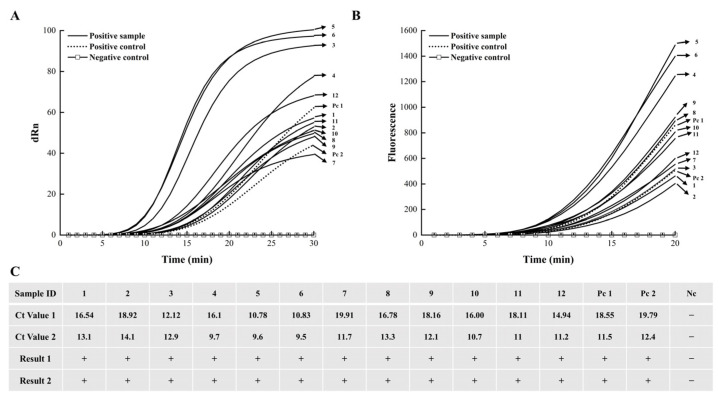
Field Evaluation Results of the EmDEA Assay on Chestnut Blight Samples. (**A**) Amplification curves recorded on a real-time qPCR instrument. (**B**) Fluorescence kinetics recorded by portable optical isothermal device. (**C**) Ct values and qualitative results for each sample (1–12) and controls. “Ct”: cycle threshold; “Ct Value 1” is the Ct measured on the qPCR instrument; “Ct Value 2” is the Ct measured on the portable optical isothermal device. “Result 1” and “Result 2” indicate positive “+” or negative “−” calls from each platform.

In forest environments, *C. parasitica* infections in mature trees often remain inconspicuous (Figure 7B,C), with yellow-orange to reddish-brown stromatic pustules typically observable only on dead trees (Figure 7D–F). The detection capability of the designed primer–probe combination was evaluated using DNA extracted from the phloem tissues of 12 infected chestnut trees. The assays were performed using a real-time qPCR instrument and the portable optical isothermal device. On the qPCR and portable optical isothermal platforms, all 12 samples and the positive control generated the characteristic amplification curves (Figure 8A,B), with Ct values corresponding to 10–20 min (Figure 8C). The negative control remained undetectable on both platforms, demonstrating that the EmDEA assay is sensitive and reproducible under laboratory and field conditions.

## 4. Discussion

Molecular diagnosis of fungal pathogens has traditionally relied on conserved genetic markers, with the internal transcribed spacer (ITS) region being the most widely used due to its high amplification efficiency and broad taxonomic resolution [19]. However, in *C. parasitica*, ITS sequences are highly conserved across isolates, limiting their discriminatory power at the intraspecific level. Alternative loci such as *TEF-1α*, β-tubulin, and large subunit ribosomal RNA genes have increasingly been used for species differentiation and phylogenetic studies [20,21,22]. Our comparative sequence analysis confirmed that *TEF-1α* exhibits appropriate inter-strain variability in *C. parasitica*, enabling more precise molecular discrimination. Accordingly, we designed and validated primers and probes targeting the *TEF-1α* gene, achieving high specificity and robust detection of *C. parasitica*.

Current diagnostic workflows for chestnut blight typically involve several sequential steps, including fungal isolation, morphological characterization, DNA extraction, PCR amplification, and sequencing for confirmation. While these methods are considered reliable, their dependence on specialized equipment, trained personnel, and time-consuming procedures limits their practicality in field settings or resource-limited environments. Although conventional molecular assays such as PCR and nested PCR offer improved sensitivity and specificity, they are constrained by the need for sophisticated instrumentation, controlled laboratory conditions, and extended processing times. Moreover, they are not well-suited for high-throughput diagnostics, as the labor-intensive post-PCR steps limit scalability and increase the risk of laboratory contamination [23,24,25]. Real-time PCR techniques, characterized by rapidity, high specificity, and exceptional sensitivity to low DNA concentrations, have been widely recognized as effective tools for plant molecular diagnostics [26,27]. Although real-time PCR enables high-throughput sample detection, its application is restricted to laboratory settings. This limitation arises from its reliance on sophisticated equipment and stable environmental conditions, making it unsuitable for on-site or field-based diagnostics.

**Figure 9:**
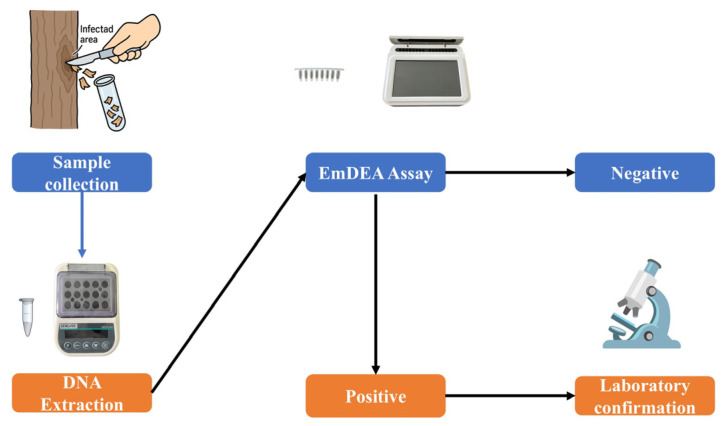
Workflow of on-site detection of chestnut blight using the EmDEA assay.

The EmDEA assay represents a valuable alternative, combining enzyme-mediated isothermal amplification with real-time fluorescence detection in a simple, rapid, and portable format. The assay can be performed using both conventional qPCR instruments and a lightweight portable optical isothermal device powered by a mobile power bank, eliminating reliance on fixed power sources. The workflow, from crude sample preparation to result readout, can be completed within 35 min, approximately one-third of the time required for conventional real-time PCR, and it requires minimal technical expertise. Additionally, the closed-tube format reduces the risk of contamination, making the system particularly suitable for field deployment in plantations, forestry stations, and remote locations (Figure 9).

While the assay demonstrates clear advantages, its field validation in this study was limited to twelve symptomatic phloem tissue samples collected from two provinces in China. To assess the conservation of primer–probe binding sites, available *TEF-1α* sequences from NCBI representing isolates from China and other regions were analyzed, and no polymorphisms were detected in these sequences. However, the limited number of sequences precludes full assessment of global genetic diversity. Moreover, minor nucleotide variations have been reported in GenBank entries at or near primer–probe binding regions. Although these differences may represent rare variants or sequencing artifacts, their potential effect on amplification efficiency was not evaluated in this study and should be considered in future work. Testing templates carrying such variants would help to further verify assay robustness. Future studies will expand field evaluation to include (i) a larger set of samples from multiple regions and countries, (ii) diverse host species and varying symptom severity, and (iii) systematic assessment of potential inhibitors in bark tissue, optimizing sample preparation to mitigate their effects. Bark tissue contains compounds such as polyphenols and tannins that may inhibit DNA amplification, potentially affecting assay sensitivity or signal timing. In the current samples, crude DNA extracts did not show significant inhibition, but samples with higher inhibitor content may require dilution, purification, or the use of inhibitor-tolerant reagents.

The EmDEA assay has some limitations, including the initial labor-intensive primer–probe optimization process and slightly higher per-sample reagent costs compared with conventional real-time PCR. These factors may pose challenges for large-scale deployment; however, the overall efficiency gains—including rapid turnaround, minimal infrastructure requirements, and reduced labor—can offset higher reagent costs. Practical considerations for field adoption include ensuring sufficient portable devices, reliable power sources, consistent reagent supply, and minimal personnel training to guarantee proper sample handling and assay performance.

Looking forward, the EmDEA platform can be further developed for multiplex detection of multiple chestnut pathogens or other economically important fungal diseases, expanding its utility in comprehensive plant health monitoring. Integration with automated sample preparation and data analysis tools would further facilitate adoption by forestry professionals and plant pathologists. By combining molecular accuracy with field accessibility, the EmDEA-based detection system bridges a crucial gap in chestnut blight diagnostics, providing an effective tool for timely disease surveillance and management.

## 5. Conclusions

In this study, we developed a novel EmDEA-based assay for rapid detection of *C. parasitica* in both cultured isolates and symptomatic *Castanea mollissima*. This single-tube, closed-format method requires minimal equipment, delivers results in under 35 min, and achieves a detection limit of 10 pg DNA per reaction with high specificity. Its portability and simplicity make it well-suited for field surveillance and quarantine enforcement. Future work will focus on expanding validation across broader regions and host species and adapting the platform for additional pathogens via probe and primer replacement.

## Figures and Tables

**Table 1:** Information of tested strains.

No.	Scientific Name	Source	Host	Detection Result
Cp1	*Cryphonectria parasitica*	Shanxi, China	*Castanea mollissima*	+
Cp2	*Cryphonectria parasitica*	Zhejiang, China	*Castanea mollissima*	+
Gd	*Gnomoniopsis daii*	Shandong, China	*Castanea mollissima*	−
Nc	*Neopestalotiopsis clavispora*	Zhejiang, China	*Castanea mollissima*	−
Nc-1	*Neopestalotiopsis clavispora*	Shanxi, China	*Castanea mollissima*	−
Nc-2	*Neopestalotiopsis clavispora*	Shandong, China	*Castanea mollissima*	−
Lt	*Lasiodiplodia theobromae*	Shandong, China	*Castanea mollissima*	−
Dh	*Diaporthe hongkongensis*	Zhejiang, China	*Castanea mollissima*	−
Cj	*Cryphonectria japonica*	Beijing, China	*Quercus* sp.	−
Cj-1	*Cryphonectria japonica*	Shandong, China	*Quercus* sp.	−
Cq1	*Cryphonectria quercicola*	Shanxi, China	*Quercus* sp.	−
Cq2	*Cryphonectria quercus*	Shandong, China	*Quercus* sp.	−
Cf	*Colletotrichum fructicola*	Zhejiang, China	*Citrus reticulata*	−
Cs	*Colletotrichum siamense*	Zhejiang, China	*Ilex chinensis*	−
Lp	*Lasiodiplodia pseudotheobromae*	Zhejiang, China	*Torreya grandis*	−
Bc	*Botrytis cinerea*	Zhejiang, China	*Vitis vinifera*	−
Aa	*Alternaria alternata*	Zhejiang, China	*Juniperus chinensis*	−
Pc	*Phytophthora cinnamomi*	Zhejiang, China	*Carya cathayensis*	−
Pv	*Pythium vexans*	Zhejiang, China	*Carya cathayensis*	−
Bd	*Botryosphaeria dothidea*	Zhejiang, China	*Carya cathayensis*	−
Bd-1	*Botryosphaeria dothidea*	Zhejiang, China	*Castanea mollissima*	−
Fo	*Fusarium oxysporum*	Zhejiang, China	*Carya cathayensis*	−
Fo-1	*Fusarium oxysporum*	Zhejiang, China	*Camellia oleifera*	−
Fo-2	*Fusarium oxysporum*	Shanxi, China	*Castanea mollissima*	−
Fg	*Fusarium graminearum*	Zhejiang, China	*Carya cathayensis*	−
Fs	*Fusarium solani*	Zhejiang, China	*Carya cathayensis*	−
Fs-1	*Fusarium solani*	Zhejiang, China	*Torreya grandis*	−
Fs-2	*Fusarium solani*	Shanxi, China	*Castanea mollissima*	−

−: negative, +: positive.

**Table 2:** Primer and probe sequences.

Primer and Probe	Name	Sequence (5′→3′)
Forward primer	F1	AAGCTAATACGACTCACTATAGGGTTAATCTCCTCCCCATCCCCAAATTCAA
	F2	AAGCTAATACGACTCACTATAGGGTCCTCCCCATCCCCAAATTCAACTTCCC
	F3	AAGCTAATACGACTCACTATAGGGCCATCCCCAAATTCAACTTCCCATACCA
	F4	AAGCTAATACGACTCACTATAGGGCCAAATTCAACTTCCCATACCATCAGCA
	F5	AAGCTAATACGACTCACTATAGGGTCAACTTCCCATACCATCAGCAGCGGCT
	F6	AAGCTAATACGACTCACTATAGGGTCCCATACCATCAGCAGCGGCTGCGCTG
Reverse primer	R1	TGCTGAACCCCGCACCAAAAAAATACAA
	R2	GAGCGAAACCGTTGCTGAACCCCGCACC
	R3	AACCGTTGCTGAACCCCGCACCAAAAAA
	R4	AGATAAGAGCGAAACCGTTGCTGAACCC
	R5	TATCTGAGATAAGAGCGAAACCGTTGCT
	R6	AAACCGTTGCTGAACCCCGCACCA
RNA probe	RNA1	FAM-GCAGCCGCUGCUGAUGGUAUGGGAAGUU-BHQ1
	RNA2	FAM-AACACAGCGCAGCCGCUGCUGAUGGUAU-BHQ1
	RNA3	FAM-UGAGACAGAACACAGCGCAGCCGCUGCU-BHQ1
	RNA4	FAM-CAGACGACUGAGACAGAACACAGCGCAG-BHQ1
	RNA5	FAM-AAACAGGGCAGACGACUGAGACAGAACA-BHQ1
	RNA6	FAM-GUUGGGCGAAACAGGGCAGACGACUGAG-BHQ1

## Data Availability

The datasets generated and/or analyzed during the current study are available for consultation upon request from the corresponding author.

## Abbreviations

The following abbreviations are used in this manuscript:

EmDEA	enzyme-mediated duplex exponential amplification
